# Primary extragastrointestinal stromal tumors of the prostate: A case report and literature review

**DOI:** 10.3389/fonc.2022.1038853

**Published:** 2022-11-08

**Authors:** Yuxuan Yang, Dengshun Sun, Kun Tang

**Affiliations:** ^1^ Department of Urology, Tongji Hospital, Tongji Medical College, Huazhong University of Science and Technology, Wuhan, China; ^2^ Second Clinical College, Tongji Medical College, Huazhong University of Science and Technology, Wuhan, China; ^3^ Department of Pathology, Tongji Hospital, Tongji Medical College, Huazhong University of Science and Technology, Wuhan, China

**Keywords:** prostate, extragastrointestinal stromal tumors, literature review, case report, imatinib

## Abstract

Gastrointestinal stromal tumors (GISTs) are the main stromal tumors of the digestive tract. Extragastrointestinal stromal tumors (EGISTs) typically originate outside the gastrointestinal tract; are not associated with the stomach or intestinal walls; and are mainly derived from the mesentery, peritoneum, posterior peritoneum, bladder, and scrotum. However, EGISTs from the prostate are rare. Here, we present a case of EGIST that passed off in the prostate of a 62-year-old man. The patient undergoes transrectal guided trans-perineal prostate puncture, and pathological reports suggest a GIST. Tumor cells are spindle-shaped, and no obvious neoplastic necrosis is seen in the sections. Immunohistochemical results are robustly positive for CD117, DOG-1, and CD34 expression. The patient had a good prognosis after treatment with imatinib, no recurrence and no metastases after six months of follow-up, and the prognosis was good. This article also provides a literature review and discussion of the treatment of EGISTs.

## Introduction

We report a case of extragastrointestinal stromal tumor (EGIST) and review the literature. The patient supplied written knowledgeable consent for the guide of this manuscript and any identifying photos or facts.

The concept of gastrointestinal stromal tumors (GISTs) was first proposed by Mazur in 1983 (1), and this is a class of tumors that originate in the interlobe tissue of the gastrointestinal tract, whose incidence accounts for 4% to 7% of celiac soft tissue tumors (2). A small proportion of nonepithelial tumors that begin outside the gastrointestinal tract, approximately 5% (3), together with the peritoneum and mesentery, are referred to as EGISTs. EGISTs and GISTs are similar in immunohistochemical phenotype and histomorphology, but EGISTs have a stronger invasive ability, and the recurrence rate after surgical resection of lesions is higher, but there is little lymph node metastasis and rarely metastasis to the lungs or other extra-abdominal organs. Our hospital admitted one case of Primary EGISTs of the prostate; present cases were reported and reviewed in conjunction with the literature.

## Case report

**Figure 1:**
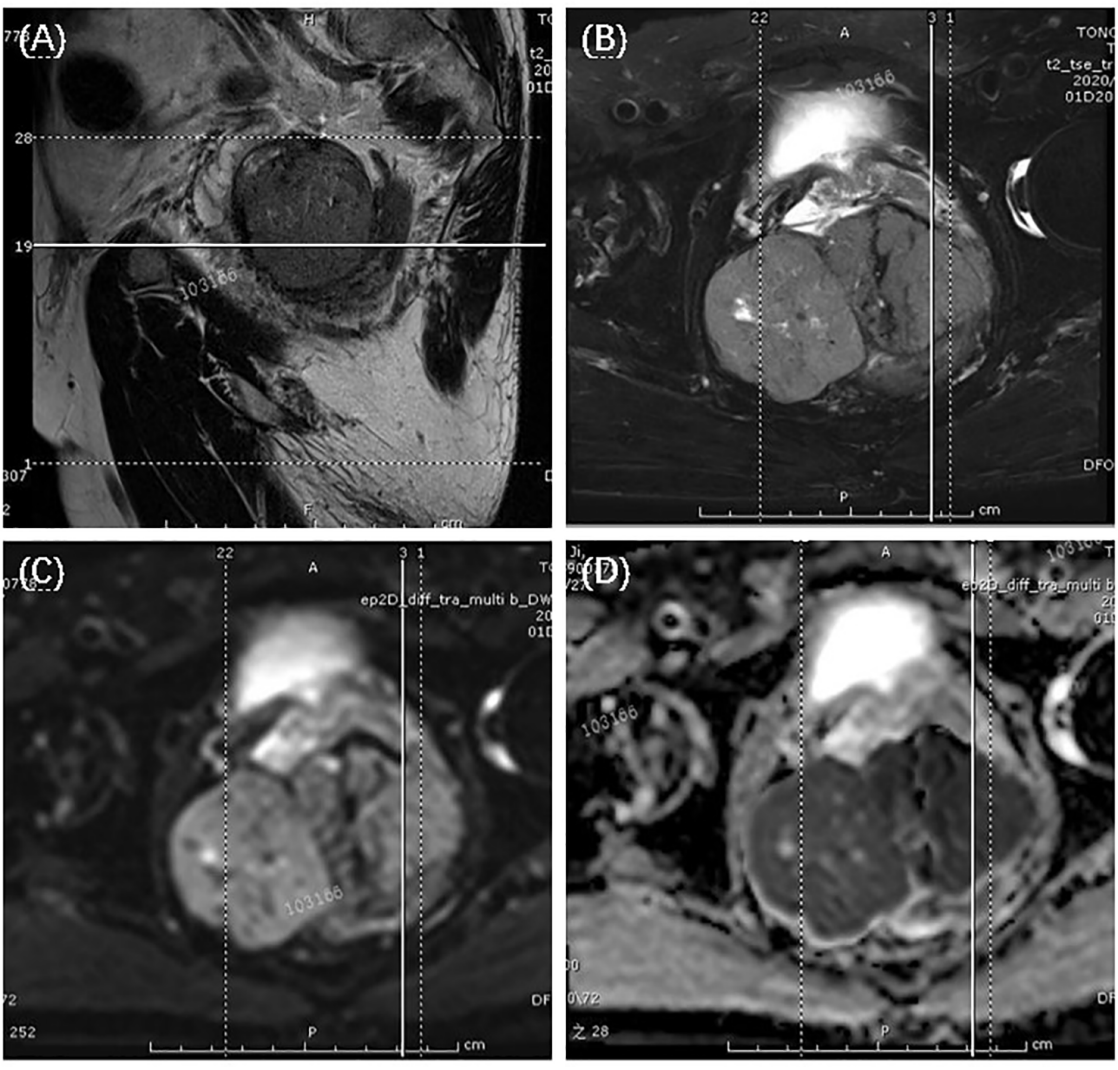
T2-weighted magnetic resonance imaging showed a large uneven tumor mass at the prostate with an unclear border with the seminal vesicles and anterior rectum, tumor appeared hyper-intensive. The arrows suggested the tumor. **(A)** sagittal view; **(B)** transection view; **(C)** diffusion-weighted imaging (DWI); **(D)** apparent diffusion coefficient (ADC) maps.

**Figure 2:**
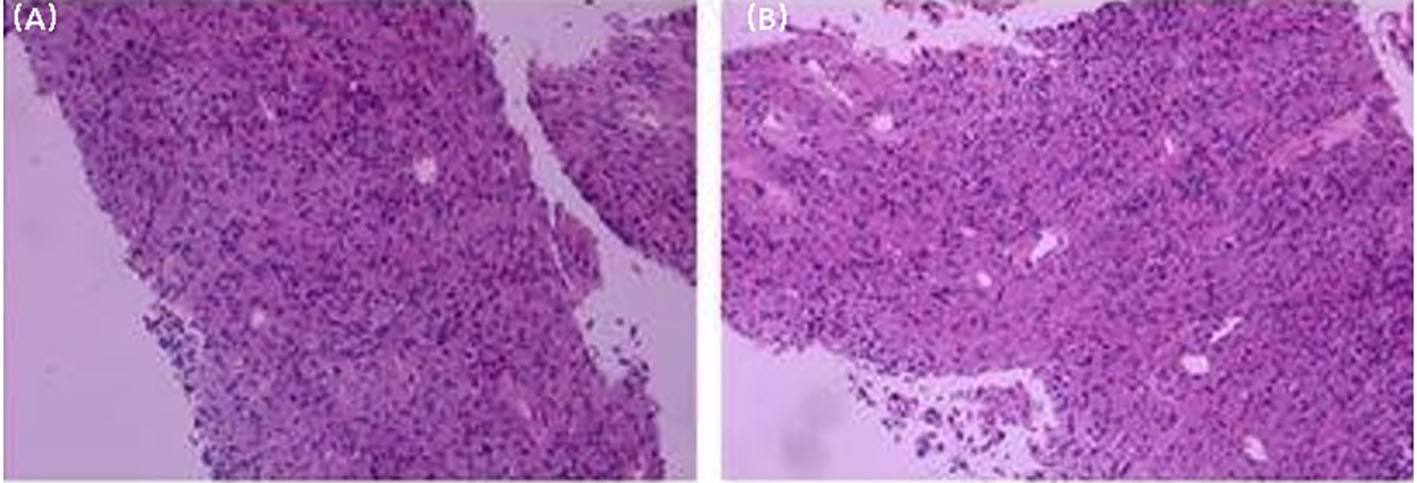
Histological microscopic examination of pathological biopsy showed that no obvious necrosis was found in the tumor, and the tumor cells were mainly spindle cells. (Original magnification: **(A)**, ×50; **(B)**, ×100).

**Figure 3:**
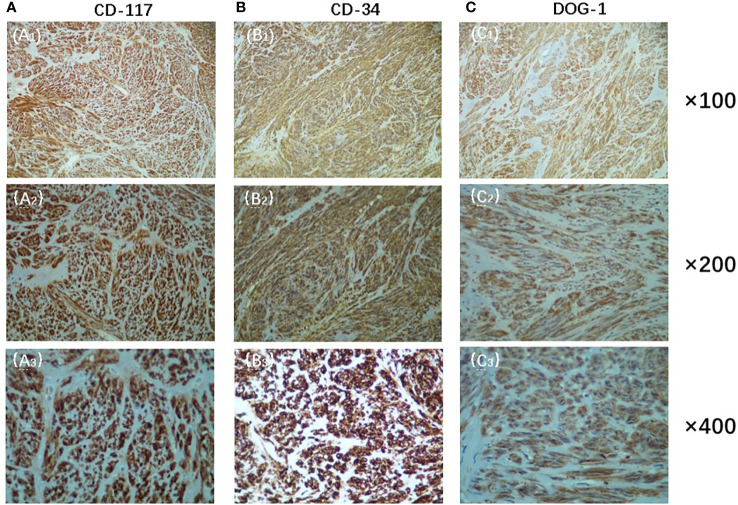
Immunohistochemistry showed positive expression of CD-117**(A)**, CD-34**(B)** and DOG-1**(C)**(Original magnification: **(A_1_)**, **(B_1_)**, **(C_1_)**, ×100;**(A_2_)**, **(B_2_)**, **(C_2_)**, ×200;**(A_3_)**, **(B_3_)**, **(C_3_)**, ×400).

The patient is a male, 62 years old, with changes in urination habits, frequent urination, and difficulty defecating for 6 months. The patient has complained of difficulty defecating without obvious precipitating causes since December 2019, and magnetic resonance imaging (MRI) checked at the local hospital suggested a prostate occupancy. The serum prostate-specific antigen (PSA) density was 1.459 ng/mL, and the carcinoembryonic antigen (CEA) density, in addition to other laboratory values, had been inside the ordinary degrees. On June 6, 2020, MRI showed irregular and large mass lesions in the prostate area, about 95×48×95 mm in size with mixed long T1 and T2 signal changes, unclear edges, unclear demarcation with the anterior wall of the seminal vesicle glands and rectum (**Figures 1A, B**), and this was accompanied by DWI high signal, ADC low value (**Figures 1C, D**), considering malignant neoplastic lesion, the possibility of prostate source, the anterior wall of the rectum and the possibility of involvement of the seminal vesicle glands (4). There were no significant abnormalities on systemic bone scans. The patient had a guided transctumal trans-perineal prostate puncture on June 8, and pathological reports suggested a GIST. Prostate tissue and significant neoplastic necrosis were not seen in each section, and tumor cells were mainly spindle-shaped (**Figures 2A, B**). Immunohistochemistry: CD-117 (+) (**Figures 3A_1_–A_3_**), CD-34 (+) (**Figures 3B_1_-B_3_**), DOG-1 (+) (**Figure 3C_1_–C_3_**), SMA (weak +), S⁃100 (-), DES (⁃),PCK(-), Ki-67 (5%). Due to the patient’s private motives, the patient refused radical prostatectomy and received only imatinib treatment. At a six-month follow-up, the prognosis was good. The sum of the length and diameter of the patient’s target lesions increased by less than 20% compared with the previous one, and no new lesions appeared, indicating that, according to the RECIST score (5), the patient’s condition changed from progressive disease (PD) to stable disease (SD). With the treatment of imatinib and tamsulosin hydrochloride, the symptoms of dysuria in the patient improved, and the tumor did not significantly enlarge or metastasize.

## Discussion

Tumors that rarely occur outside the gastrointestinal tract (5%) are called EGISTs (6), and clinical pathology shows that extraterritorial stromal tumors often originate from the mesentery, peritoneum, posterior peritoneum, scrotum, bladder, ovaries, pancreas, and vagina (3), of which extraterrestrial stromal tumors from the prostate are very rare. In previous reports, the main clinical manifestations of patients with primary EGIST of the prostate include urinary frequency, urgency, dysuria, acute urinary retention, vague perineal pain, and constipation or a combination of one or more of these symptoms (7). Compared with other GISTs, EGIST masses can grow to a larger volume in the underlying peritoneal cavity and retroperitoneal space, so patients with EGIST are not easy to detect early and need to be judged by imaging techniques. MRI and CT are the most common imaging tests of EGIST. MRI can not only provide a clear image of tumor growth and adjacent tissues, but also distinguish whether the tumor has hematoma, necrosis, and tissue good and evil properties to decide the diploma of adhesion with surrounding tissues (such as rectum) (8). PET/CT can sensitively observe the local situation of the tumor and also can understand the systemic tumor invasion and metastasis. Development of high-risk EGIST treatment has guiding significance. The clinical symptoms of this case have been present for more than half a year, and MRI shows that the lesion is large in size and there is necrosis inside the lesion, which is consistent with the imaging performance of stromal tumors.

EGIST requires a differential diagnosis with pelvic, retroperitoneal sources of malignancies, such as liposarcoma, lymphoma, leiomyoma, etc. This case originated in the prostate gland and presents as a large cystic tumor of the prostate gland, which requires differential diagnosis with malignant tumors such as prostate cancer. Unlike prostate cancer, which often occurs in the elderly, EGISTs, which originate in the prostate, can also be seen in young adult patients and do not have obvious age characteristics. Moreover, the clinical symptoms of EGISTs that originate in the prostate are often accompanied by problems with the digestive tract system, such as poor bowel movements and tenesmus. A pathological puncture biopsy can help us identify EGISTs. Among cell types, spindle cells are the most common cell type in EGIST tissues, accounting for about 70% of the number of cells in EGIST tissues. The cells are spiral-shaped, cytoplasmic clumps, reddish, the membrane is not clear, even stained, and the nucleus is not clear. In immunohistochemistry, CD117 is a fabrication from the c-kit proto-oncogene as a transmembrane receptor egg white of tyrosine kinase, and it is one of the extra unique antibodies to diagnose EGISTs (9). Moreover, as a highly glycosylated type I transmembrane glycoprotein, CD34 can participate in the transport and colonization of hematopoietic stem cells, and its expression rate in EGISTs is 50% to 70%. The combined detection of CD117 and CD34 can reduce the missed and false positives of EGISTs. In addition, the overall sensitivity of DOG-1 in GISTs is as high as 94.4% (10). Compared with CD117 and CD34, it has higher specificity and sensitivity in the diagnosis of EGISTs, and its positive expression can also be used as an important indicator to distinguish GISTs. Based on the positive rates of CD34, CD117, and DOG-1 in the patient’s immunohistochemistry, we established the diagnosis of EGIST in this case. Moreover, the maximum tumor size of this case is 9.5 cm, and the mitosis rate is <5/50 HPF; according to the GIST risk level grading standard (11) issued by the National Institutes of Health in 2008, the case belongs to the intermediate risk group.

We conducted a pooled analysis of eight cases of EGIST patients originating in the prostate and our case (**Table 1**) with an average age of 53.8 years (range, 31–67 years) and an average tumor size of 12.075 cm (range, 6–28 cm). According to the GIST risk level grading standard (11), 4/8 patients (50%) were medium-risk and 4/8 patients (50%) were high-risk. The mean follow-up period was 9.1 months (range, 3–19 months), and none of the remaining seven patients showed recurrence or metastasis (data were not collected in one patient) except for one patient who died of intermittent drug use due to worsening of his condition. In addition to this, we found that all but one patient had no PSA collected; the remaining eight patients had normal levels (8/9, 88.9%), and PSA was mainly secreted by human prostate epithelial cells, which provided direction for us to make a differential diagnosis of prostate cancer and EGIST that originated in the prostate.

**Table 1:** Brief review of primary extra-gastrointestinal stromal tumors of the prostate in the literature.

Reference	Age/Sex	Clinical feature	Tumor size (cm)	PSA(ng/ml)	Tumor risk level grading*	Immunohistochemistry	Treatment	Follow-up time(Ms)	0utcomes
Jung-Sik et al. (8)(2014)	50/M	residual urine sensation, and perineal discomfort	9.7×8.8×8.4	0.85	medium risk	CD117(+) , CD34 (+) , S100 (-)	N /A	N/A	N/A
Liu et al. (11)(2014)	55/M	dysuria and urinary frequency	10×10.5×9.5	2.01	high risk	CD117 (+) , CD34 (+) , DOG-1(+), S100 (-) , SMA (-)	imstinib	12	No recurrence and metastasis
Zhang et al. (12)(2014)	31/M	intermittent gross hematuria and dysuria	6×6.1×6.5	1.1	medium risk	CD117 (+) , CD34 (+),DOG-1(+),S100 (-) , SMA (-)	imstinib	3	Dead
Etit D et al. (13)(2018)	56/M	anus pain	6	1.1	medium risk	CD117 (+) CD34 (+) DOG-1 (+) S100 (-) , SMA (-)	RP+imatinib	6	Decreased tumoral volume
Haixing et al. (14)(2019)	43/M	no obvious symptoms	13×10×16	2.7	high risk	CD117 (+) CD34 (+), DOG-1(+) , S100 (-) SMA (-)	PR-imstinib	6	No recurrence and metastasis
Gaurav Garg et al. (15)2019)	55/M	lower urinary tract symptoms	N/A	3.2	N/A	CD117(+), CD34 (+) , S100 (-)	imstinib	12	Decreased tumoral volume
Lu et al. (16)(2021)	65/M	intermittent hematuria and lower urinary tract symptoms	10.4×8.6×8	N /A	high risk	CD117 (+) , CD34 (+), DOG-1 (+) , S100 (-) , SMA (-)	RP+imatinib	19	No recurrence and metastasis,with elevated PSA
Dilasma et al. (18)(2021)	67/M	vague abdominal pain, discomfort and sudden reduction of appetite	28×23×16	N/A	high risk	CD117 (+) , CD34 (+) , S100 (-). , SMA (-)	RP	N/A	No recurrence and metastasis
Present Case	62/M	changes in urination habits, frequent urination, and difficulty defecating	9.5×4.8×9.5	1.459	medium risk	CD117 (+) , CD34 (+) , DOG-1(+), S100 (-) , SMA (-)	imatinib	6	No recurrence and metastasis

N/A, not available; PSA, prostate specific antigen; RP, radical prostatectomy.

*: issued by the National Institutes of Health in 2008.

Nowadays, treatment modalities for EGISTs typically include surgery, medication, and other treatments. However, since EGISTs are not sensitive to traditional chemotherapy drugs (19), the traditional treatment of EGISTs is still surgical resection. The most common form of surgical treatment is radical prostatectomy, which involves total, open, laparoscopic or robot-assisted surgery to remove the entire prostate gland and seminal vesicles (20). However, an EGIST tumor is generally brittle and larger, and it is easy to rupture the tumor body and lead to the implantation of the tumor during the operation, so it is necessary to minimize touching the tumor during the operation, and the technique should be gentle to prevent the tumor rupture from causing abdominal implantation and minimize the spread as much as possible. Because less than 10% of patients present with lymphatic metastases (17), extensive lymph node dissection is not recommended. Moreover, EGISTs have a poor prognosis compared with prostate cancer and are usually combined with imatinib for adjuvant therapy. If the patient has a poor prognosis, relapses, and systemic metastases, surgery is a greater risk and ineffective, so it is generally not considered, and targeted therapy with a tyrosine kinase inhibitor (imatinib mesylate) is usually chosen to control the lesion (13). For patients with local recurrence, we can do an en-bloc resection of the tumor with a macroscopically negative margin followed by adjuvant imatinib (21). For patients who develop imatinib resistance, we can use second-line sunitinib for treatment, but other treatment options after imatinib resistance are not ideal. In the case of sunitinib, for example, the adverse effects are relatively large, such as heart disease, uncontrollable hypertension, and hypothyroidism (22).

## Conclusion

EGISTs that originate within the prostate are rare and tough to detect at an early stage. Without adequate case knowledge and adjunctive examinations, preoperative diagnosis of patients is difficult. Due to its special origin, closely associated with the prostate, tumors usually develop in middle and old age, accompanied by symptoms of frequent urination with a poor prognosis and require differential diagnosis with retroperitoneal, pelvic-derived malignancies. Diagnosis depends primarily on the positive rates of CD117, DOG-1, and CD34 in immunohistochemical results. Prostate masses are also monitored for differential diagnosis with the help of pathological biopsy and imaging. Radical prostatectomy is the maximum dominant treatment, and adjuvant therapy can be combined with imatinib in the later stages. In addition, in some cases, patients with intermediate and advanced stages are unable to undergo surgical resection therapy, and conservative treatment with imatinib alone is considered. However, sometimes, patients choose to abandon surgical excision treatment for financial reasons and consider imatinib alone for conservative treatment. The patient in this case is of this type, and this type of patient needs attention to be paid.

## Data availability statement

The data sets for this article are not publicly available due to concerns regarding participant/patient anonymity. Requests to access the datasets should be directed to the corresponding author.

## Ethics statement

Written informed consent was obtained from the individual(s) for the publication of any potentially identifiable images or data included in this article.

## Author contributions

YY and KT conceived the idea for the article and designed the case report. YY reviewed the literature and drafted the article. KT and DS prepared radiological and histology figures and provided immunohistochemical analysis. All authors contributed to the article and approved the submitted version.

## Funding

This study was supported by the National Natural Science Foundation of China (No.81900645, No.82270804), 2019 Wuhan Yellow Crane Talent Program (Outstanding Young Talents), and the Tongji Hospital (HUST) Foundation for Excellent Young Scientist (No.2020YQ15).

## Conflict of interest

The authors declare that the research was conducted in the absence of any commercial or financial relationships that could be construed as a potential conflict of interest.

## Publisher’s note

All claims expressed in this article are solely those of the authors and do not necessarily represent those of their affiliated organizations, or those of the publisher, the editors and the reviewers. Any product that may be evaluated in this article, or claim that may be made by its manufacturer, is not guaranteed or endorsed by the publisher.
